# A Security Framework for Increasing Data and Device Integrity in Internet of Things Systems

**DOI:** 10.3390/s23177532

**Published:** 2023-08-30

**Authors:** Amir Dirin, Ian Oliver, Teemu H. Laine

**Affiliations:** 1 Department of ICT, Metropolia University of Applied Sciences, 00920 Helsinki, Finland; amir.dirin@metropolia.fi; 2 Nokia Bell Labs, 02610 Espoo, Finland; ian.oliver@nokia.com; 3 Department of Digital Media, Ajou University, Suwon 16499, Republic of Korea

**Keywords:** Internet of Things, security, data integrity, device integrity, framework, proof-of-concept

## Abstract

The trustworthiness of a system is not just about proving the identity or integrity of the hardware but also extends to the data, control, and management planes of communication between devices and the software they are running. This trust in data and device integrity is desirable for Internet of Things (IoT) systems, especially in critical environments. In this study, we developed a security framework, IoTAttest, for building IoT systems that leverage the Trusted Platform Module 2.0 and remote attestation technologies to enable the establishment of IoT devices’ collected data and control plan traffic integrity. After presenting the features and reference architecture of IoTAttest, we evaluated the privacy preservation and validity through the implementation of two proof-of-concept IoT applications that were designed by two teams of university students based on the reference architecture. After the development, the developers answered open questions regarding their experience and perceptions of the framework’s usability, limitations, scalability, extensibility, potential, and security. The results indicate that IoTAttest can be used to develop IoT systems with effective attestation to achieve device and data integrity. The proof-of-concept solutions’ outcomes illustrate the functionalities and performance of the IoT framework. The feedback from the proof-of-concept developers affirms that they perceived the framework as usable, scalable, extensible, and secure.

## 1. Introduction

Academics and professionals are increasingly interested in the potential of the Internet of Things (IoT), and it has become popular across various domains and industries, including healthcare [1], education [2], and consumer electronics [3]. With the increasing use of interconnected devices, the likelihood of intrusions and security breaches has also grown. As a result, it is crucial to establish a framework that safeguards the security of IoT systems against potential threats. Given that IoT systems are connected to the internet, they are susceptible to the same security risks as other connected devices, such as cyberattacks and malware attacks. This study presents the implementation and validation of IoTAttest, an IoT security framework for ensuring the integrity of devices and data in IoT systems, which is contingent on the security and integrity of its components. It is imperative to authenticate the system’s components in advance to ensure their validity, especially when the data source is unfamiliar to the system.

The objective of this paper is to showcase the functionality of IoTAttest with an emphasis on data integration and privacy preservation among diverse IoT devices and clients. Many IoT evaluations conducted in research were focused on IoT security, sensor networks, and IoT management [4]. Data integrity between different devices is important to investigate in IoT systems [5] because otherwise the IoT systems cannot reach a sufficient level of trustworthiness among users. The assessment of IoTAttest focuses on two proofs of concept (PoC), including a scenario with an embedded system where multiple sensors collaborate to accomplish a task (PoC1), and a scenario based on a real-world environment where resources are shared, the number of clients vary, and delivery is tailored according to individual client requirements (PoC2).

By implementing IoTAttest, real-time monitoring of the validity and reliability of collected data becomes achievable. Furthermore, the framework streamlines the process of expanding the IoT system by enabling the effortless addition and removal of additional components while preserving privacy and ensuring integrity. The privacy preservation and validity of IoTAttest are assessed through two PoC implementations related to sensor device management and train information display management, respectively. Additionally, both PoCs were subjected to testing that simulated intrusion mechanisms to verify that the framework detects unauthorized devices and tampered data, thus rejecting them.

## 2. Background

IoT security research is constantly evolving, as is the rise in cyber threats and cyberattacks. A wide range of cyber security domains are involved in the IoT ecosystem, such as privacy, confidentiality, user security, security of system infrastructures, data, and devices, as well as ensuring the functionality and availability of services built upon IoT [6]. Moreover, IoT security concerns different layers of the system, including hardware, software, and network security. 

IoT security holds significant importance within the IoT research community for many reasons. Insufficient security measures in IoT systems can make devices, networks, and data vulnerable to unauthorized entities. In typical IoT system setups, various interconnected devices facilitate the exchange of substantial volumes of data, necessitating protection from unauthorized exposure. This underscores the importance of safeguarding data privacy [7], encompassing personal, financial, and health-related information, against both physical and cyber threats [8]. 

Moreover, the susceptibility of hardware and devices to physical attacks presents a distinct challenge that IoT systems must proactively address. The ramifications of inadequate security within IoT systems extend to economic implications, erosion of user trust, user safety, protection of critical infrastructure, and the need for ongoing device and system maintenance to pre-emptively manage potential failures. Furthermore, an additional hurdle that requires accurate design and development in IoT systems is the precision of the data and the guarantee of accurate information from various sources [9]. 

In the following sections, we review previous work on IoT security related to the proposed IoT security framework.

### 2.1. Hardware Component Security in the IoT Context

IoT systems are composed of various hardware components, such as sensors, controllers, input and output devices, and peripheral devices. These hardware components are interconnected and work together to perform the required functions. In the event of an intrusion or malfunctioning of any of these hardware components, it will impact the performance and reliability of the whole system. Therefore, the security of the hardware components also contributes to the overall security of the system. In the IoT context, hardware security is important to ensure the security of data. There are several ways to ensure the hardware security of IoT components, such as continuous remote firmware updates of IoT components. Furthermore, hardware security is also associated with hardware-based authentication, for example, through a Trusted Platform Module (TPM) chip based on the ISO/IEC 11889-1:2015 standard [10] or including a secure boot process such as using cryptographic techniques to verify the authenticity of the firmware. Other examples of hardware security techniques include the Trusted Execution Environment (TEE) in the processor, which enables the processing of cryptographic keys and other critical data in a protected area [11], and Physical Tamper Resistance chips [12] that ensure the security of hardware devices for physical attacks. The anti-tamper sensor ensures detection and response to physical tampering attempts. 

### 2.2. Software Security in the IoT Context

Software security is critical to ensuring the integrity of any computer system, and it refers to designing and implementing the software to prevent malicious attacks and react to them, for example, through real-time monitoring, incident responses, and forensic analysis [13]. In the IoT context, the software is more vulnerable to security threats due to the interconnected devices, extensibility of the system, and difficulty of continuous monitoring. Xu et al. [14] identified the software security scope for IoT as trusted sensing, computation, communication, privacy, and digital forgetting. In another study, Hiromoto et al. [15] proposed a cyber-secure, IoT supply chain risk management architecture to reduce vulnerabilities by applying machine learning, cryptographic hardware monitoring, and distributed system coordination. Their proposed system aims to reduce the risks associated with malicious actors in supply chains. 

Any secure software needs to be carefully designed and undergo various testing processes. Software security ranges from local implementation errors to interprocedural interface errors and design-level mistakes. Among various approaches, authentication and authorization have been common approaches to ensuring the security of IoT systems [16]. Only authorized devices should be able to connect, receive, and transfer data. Encryption is yet another commonly used software and data security technique in which all communication between IoT devices, cloud platforms, and gateways is encrypted to prevent unauthorized access to the data [17,18,19]. In contemporary IoT systems, blockchain technology [20,21] has been commonly used to enhance the security of IoT systems. Blockchain brings several benefits to the IoT system, as it ensures the integrity, immutability, and transparency of the data in the IoT system. Additionally, blockchain networks have used distributed consensus algorithms such as proof of work (PoW) or proof of stake (PoS) [22] to validate the state of the blockchain. 

### 2.3. Security and Privacy Threats in the IoT Context

Within the context of IoT, the term “intrusion” denotes the activities of unauthorized individuals, entities, or devices that exert an adverse influence on the performance of the IoT system. Such intrusions may entail the exertion of control over the devices or the manipulation of networks by malicious actors, thereby compromising the system’s integrity [23]. Potential IoT intrusions that may occur in IoT systems are unauthorized access to connected devices, exploitation of vulnerabilities in IoT devices or networks, and malware and ransomware attached to IoT devices or networks [24,25]. Other attacks include Denial of Service (DoS) and Distributed Denial of Service (DDoS) attacks that overwhelm IoT networks [26,27], sniffing attacks that intercept and monitor data transmissions between IoT devices or between devices and the network [26], data injection attacks that insert malicious data into the IoT system, and physical tampering with IoT devices or systems to gain unauthorized access or control [28].

Sharing data with different entities from IoT devices is at the core of many IoT systems. This includes sharing the device’s state and collected data with remote parties. The privacy of data during attestation and data delivery is a challenge for which various solutions are recommended. For example, Xu et al. [29] proposed a remote attestation model based on a privacy-preserved blockchain model. In addition, Larsen et al. [30] recommended a system comprising trusted computing elements on a direct anonymous attestation using TPM. Aligned with this, Xiong et al. [31] suggested a scalable network attestation schema for simultaneously increasing security and privacy preservation.

### 2.4. Device and Data Integrity in the IoT Context

Device and data integrity are vital aspects of digital solutions, ensuring the reliability of data exchanged between different components within a system. Security considerations are the foundation of any IoT system [32]. In the realm of IoT, device and data integrity are particularly crucial and should be considered during the design phase. Device integrity refers to safeguarding the connected hardware and software components against unauthorized modifications or tampering, e.g., launching attacks, or hacking. Data integrity refers to ensuring that the data are accurate and consistent, and remain intact throughout the system. Loi et al. [33] divided device and data integrity into four categories: confidentiality, integrity, access control of the IoT device, and reflective attacks that can be launched from within IoT devices. 

### 2.5. IoT Security Frameworks

Multiple IoT security frameworks have been proposed by key players in the field. These frameworks aim to provide comprehensive guidelines and best practices for ensuring the security of IoT devices, networks, and data. One of the most notable and popular IoT security frameworks is AWS IoT (Amazon Web Services) IoT [34], which is a cloud platform for IoT [35]. The AWS IoT framework enables devices to connect and interact securely with different devices. ARM Mbed IoT [36] is another popular framework that provides an ecosystem to build standalone or networked IoT solutions. ARM Mbed IoT provides a scalable, connected, and secure environment for IoT devices by integrating Mbed tools and services through ARM microcontrollers (Mbed OS, Mbed Device Connector, and Mbed Connect Cloud). Azure IoT suite is yet another popular platform that enables end users to design, develop, and deploy IoT devices, exchange data, and perform data operations such as aggregation, multidimensional analysis, and transformations. Google released its own version of the IoT framework called the Brillo/Weave platform. Brillo is based on an Android-based operating system for low-power devices, and Weave is a communication shell for interactions between devices. The weave is responsible for registering a device in the cloud and enabling it to send/receive remote commands. The Google solution specifically targeted smart homes and general IoT devices. As the applications of the IoT have become ubiquitous, the security issues and interoperability challenges have also increased. Furthermore, the scalability and complexity of IoT systems mandate that they are constantly updated and improved. 

### 2.6. Framework Validation Methodology

A framework’s rigor is often assessed by ensuring its validity and reliability. Refining a framework’s functionality, performance, generalization, and usability is an important step to gain the framework’s acceptance and applicability. The methods of validation are dependent on the discipline and domains that the framework is proposed for; e.g., surveys and questions have been used in previous research [37]. In the software engineering domain, applied validity methods evolve over time, driven by the frameworks’ growing complexity resulting from technological advancements. Validation through real-world scenarios is among the common methods of framework and system validation in the context of IoT [38]. Real-world deployment scenarios provide the means to validate IoT solutions arising from the framework across diverse contexts on a large scale. Another common method for real-world validation is the PoC method, which is used to demonstrate how a proposed product concept or a conceptual framework can fulfill its requirements [39,40,41]. For example, a PoC can be constructed to pilot a software product before its actual development and before making a decision to invest [42]. A PoC can also be designed to aid in verifying certain assumptions that the solution meets its intended objectives [40]. The PoC methodology typically comprises a series of sequential stages, including problem domain delineation, formulation of a hypothesis, design of the PoC product, development of a functional PoC, rigorous testing, refinement of the PoC, and communication of results to relevant stakeholders.

## 3. Research Question and Method

### 3.1. Research Question

We formulated the following research question to be answered in this study through the development and validation of IoTAttest, the proposed IoT security framework: What are the key components necessary for ensuring data and device integrity in an IoT system?

### 3.2. Research Method

We applied the multi-proof-of-concept (multi-PoC) methodology [41] to validate the proposed framework. We validated the proposed IoT framework through the utilization of two distinct PoC projects. The framework was first introduced to two groups of four university students, who were then requested to design, implement, and test an IoT system concept using the framework during the fall of 2022 and spring of 2023, respectively. The second author of this study provided the PoC concepts and requirements. Students had a weekly meeting with the first and second authors about the project. The first author acted as a scrum master and the second author as a product owner. The project requirements and the overall concept were provided by the first author, who also provided the teams with Raspberry Pi devices, sensors, TPM modules, MQTT server, remote attestation infrastructure, and access rights to database. The projects had two main objectives: (i) to create a PoC IoT solution for a real client, and (ii) to evaluate the feasibility and validity of the framework. The teams utilized the Scrum methodology with two-week sprint periods to manage and execute the project. 

To ensure effective collaboration and communication within the development team they had daily scrum meetings, either face-to-face or via the Discord application. The two PoC projects were completed within a span of seven weeks, with the final week allocated for project documentation and the presentation of the project outcomes to the relevant stakeholders. As part of our project management approach, we utilized the Nektion (https://www.nektion.com/ accessed on 14 July 2023) tool to effectively monitor and track the utilization of time and resources, as well as to keep abreast of the implementation status throughout the project’s lifecycle.

Ensuring the project’s success from a software engineering perspective was the responsibility of the first author, while the second author evaluated and approved the PoC validation. The validation procedure was enacted subsequent to the deployment of the resulting PoC product within a real environment, wherein the test outcomes were rigorously assessed in accordance with the initial requirements. Evaluation of the PoC project outputs was conducted from both a project implementation perspective by the first author and a requirements and technology perspective by the second author. Finally, a qualitative questionnaire prepared by the first and third authors was used to perform an assessment of the framework from the perspective of developers. This questionnaire, which is show in Appendix A, was given to the developers upon completion of their projects.

## 4. Concept and Reference Architecture of IoTAttest

**Figure 1:**
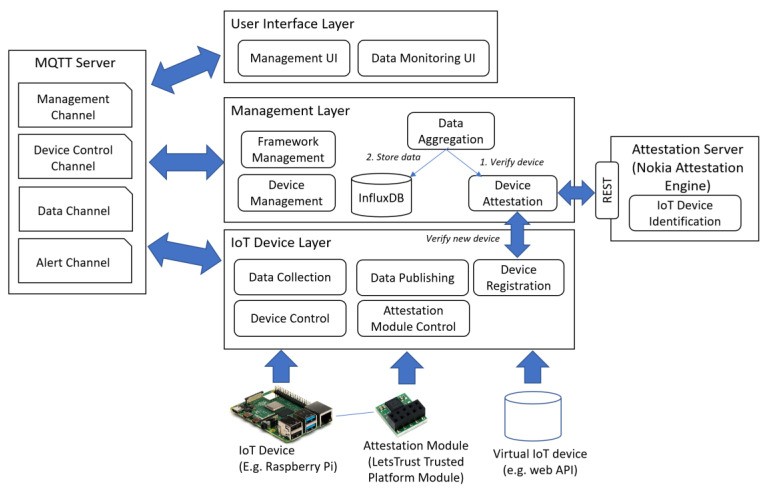
Reference architecture of the IoTAttest framework.

The proposed IoT security framework aims to enable the rapid and simplified development of sensor-driven IoT systems by orchestrating the work related to sensor data aggregation, monitoring, and attestation. The programmer can focus on adapting the framework for their needs and extending it to add more features. The IoTAttest framework loosely coupled, layered architecture is depicted in Figure 1: (i) The IoT Device Layer connects to various IoT devices, thus mediating commands and data between the devices and the Management Layer; (ii) the Management Layer includes services for overall framework management, device management, device attestation, and data aggregation; and (iii) the User Interface Layer provides tools for the user to access the framework management and data monitoring functionalities. The framework layers communicate with each other using a Message Queue Telemetry Transport (MQTT) server [43]. Moreover, the management layer communicates with an instance of an attestation server (e.g., Nokia Attestation Engine) by Representational State Transfer (REST). In the following sections, we explain each part of the framework in detail.

### 4.1. Core Packages

**Figure 2:**
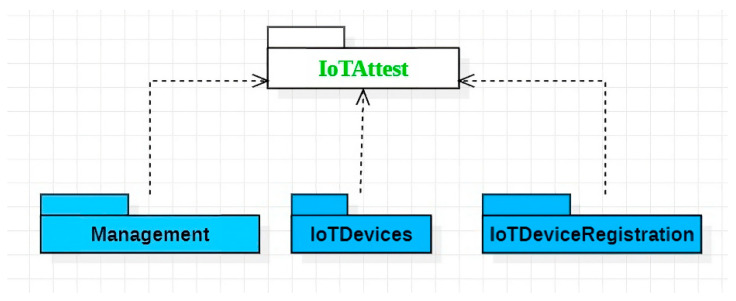
A package diagram of the key components of the IoTAttest framework.

A package diagram of the framework’s core components is presented in Figure 2. All components in the packages are configured using JSON-based configuration files, which define, for instance, available IoT devices as well as connectivity configuration to the MQTT server and attestation server. The Management package handles overall device management and uses attestation and aggregation libraries for managing sensor device validation and data aggregation, respectively. 

The IoTDeviceRegistration package comprises components that provide services for all IoT Devices, such as initializing sensor configuration based on a configuration file and publishing data to the management module via MQTT. The IoTDevices package implements the specific data collection procedure to handle data retrieval from the respective data sources (e.g., sensor) and publish the data via MQTT. The data collection procedure may include parameters such as the data retrieval interval, data pre-processing, and format conversion. Moreover, one IoT device component can acquire data from multiple physical sensors. For example, WeatherDevice can retrieve data from a temperature and a humidity sensor attached to the device. 

The IoTDevices package can contain representations of both physical IoT devices and virtual IoT devices, with the latter allowing more freedom for the developer to control data retrieval and other functionality. For example, a developer might implement a virtual sensor that retrieves data from a web API based on a logic other than simple periodic polling. 

### 4.2. IoT Device Layer

The IoT Device Layer of the platform is responsible for IoT device management, including low-level data collection and publishing operations. It can also send control messages to IoT devices, for example, to configure, pause, stop, and resume data collection. IoT devices, such as a Raspberry Pi with attached sensors, are extended with a TPM, such as Let’s Trust TPM, that has a standardized secure cryptoprocessor for implementing device-level security measures in computer systems. Most motherboards are extendable with TPM module plugins to extend the security of devices with TPM capability. The purpose of using a TPM in the proposed framework is to produce, retain, and restrict cryptographic keys, and its implementation of various physical security measures makes it resistant to tampering. TPM version 2.0 enables the implementation through chipsets, ARM SoC, and AMD Ryzen Pro CPUs. In addition, firmware TPMs, which are software solutions that can be run in a CPU’s TEE, or virtual TPMs can also be used. 

### 4.3. Management Layer

The Management Layer is mainly responsible for the components controlling the framework. The management layer consists of the following components to ensure the IoT operation, security, and efficiency of the IoT deployments:

- Framework Management provides tools, libraries, and services that assist the development of IoT solutions. The framework provides the foundation such as APIs and protocols for building applications.
- Device Management has the role of administering and controlling the IoT devices throughout their lifecycle by communicating with the IoT Device Layer. This component ensures the device provisioning, device configuration, firmware updates, and monitoring of the device performance, and device authentication and security.
- Device Attestation is a component that ensures the integrity and authenticity of IoT devices attached to the system. This includes verifying the genuineness, trustworthiness, and secure execution of the operations of the connected device. We implemented it using remote attestation [44], which is commonly applied in IoT environments. For this purpose, we utilized Nokia Attestation Engine A10, which is an open source remote attestation system (https://github.com/nokia/AttestationEngine accessed on 14 July 2023). Device attestation activities involve the device’s hardware, firmware, and software components to measure and report the current state of each device.
- Data Aggregation collects data from various IoT devices into a single data repository. Data aggregation in the proposed framework involves gathering data points, analyzing, reporting, and transforming them into a manageable format. Hence the main aim of data aggregation is to increase reliability [45].

### 4.4. User Interface Layer

The framework’s user interface components are implemented as representational state transfer (REST) services. REST is an architectural approach that provides open and widely used standards to enable computer systems to interact with each other over the web [46]. REST APIs are commonly used to build IoT systems’ user interfaces that can be accessed by any web browser, where the web client can request the server to add, update, delete, and retrieve resources. 

The Management UI component provides a web-based interface for system and device management. It can be used for monitoring, configuring, and controlling attached devices. User account management is also implemented as part of Management UI. The Data Monitoring UI provides access to collected data as well as visualizations of the data. Moreover, data searching and downloading functionality can be implemented.

### 4.5. MQTT Server

MQTT is a lightweight publish–subscribe protocol developed for efficient communication between IoT devices that have constraints in capacity, such as network bandwidth, power, or processing. It is meant to be used with systems that rely on asynchronous queue-based communication. IoTAttest uses MQTT extensively to facilitate communication between components. The MQTT server should be behind a firewall and all communications between the MQTT server and its clients should be encrypted (e.g., TLS). Various channels can be used to communicate messages within the framework. The reference architecture (Figure 1) proposes the following four channels, which can be easily extended by registering new channels to the MQTT server:

- Management channel is meant for general messages pertaining to the management of the MQTT server. The MQTT client may subscribe to topics to receive messages published by other clients on the subscribed topics. Messages can also be retained and stored by the MQTT server and shared with new subscribers. Finally, ensuring the quality of the service is one of the key tasks of the management channel.
- Device control channel relays messages related to IoT device control, such as starting, pausing, or stopping data collection on the device. Device control channel is based on a publish–subscribe model in which the control channel publishes and the IoT devices act as subscribers.
- Data channel is used for passing data from the registered IoT devices to the data aggregation component in the Management Layer. The data package is accepted only if the device that sends it has been attested.
- Alert channel is used for various alerts to be delivered in the system, such as alerts sent from the Management Layer to IoT devices, or vice versa. The MQTT server may also send alert messages via this channel.

## 5. Framework Validation by PoC Development

The framework was validated by implementing two PoCs and subsequently testing them. As per the proposed IoT security framework architecture, both PoCs involved the use of connected IoT devices coupled with sensors and the processing of data via MQTT and TPM before delivering them to output devices. Table 1 and Table 2 present the IoT devices and software that were used in the PoC1 and PoC2 projects, respectively. The IoT devices were employed based on the recommendation by the researchers and the target use cases. In the following sections, we describe the PoC systems that were developed based on the proposed IoT security framework.

### 5.1. PoC 1: Sensor Management System

**Figure 3:**
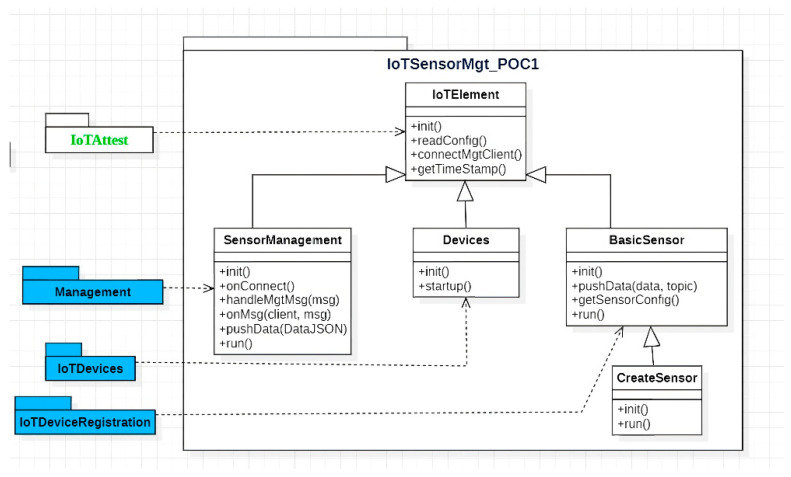
Architecture of the sensor management system.

**Figure 4:**
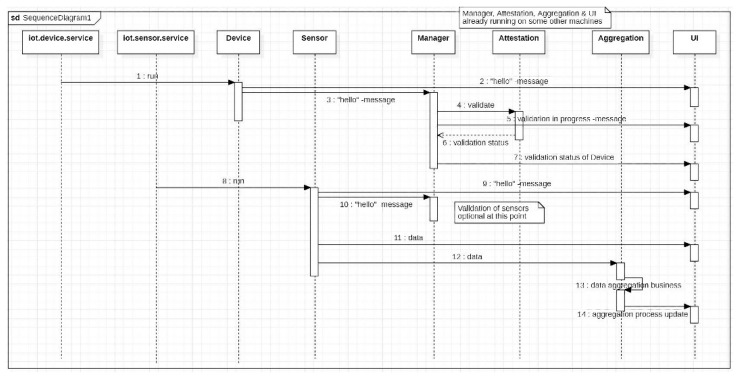
Sequence diagram of the sensor management system.

The overall goal of the first PoC application, a management system for IoT sensor devices, was to create some sensor information flows and display them on a dashboard. Figure 3 depicts the architecture of the application whereas the sequence diagram of the system operation is visualized in Figure 4. The sensor management system was built with user interfaces for viewing the status of sensor devices and the data sent by them. Each IoT sensor device was equipped with a TPM component that was used for attestation. Moreover, the devices communicated with the Management Layer using MQTT channels. The sensors implemented in the PoC were humidity, infrared, and time of flight. 

**Figure 5:**
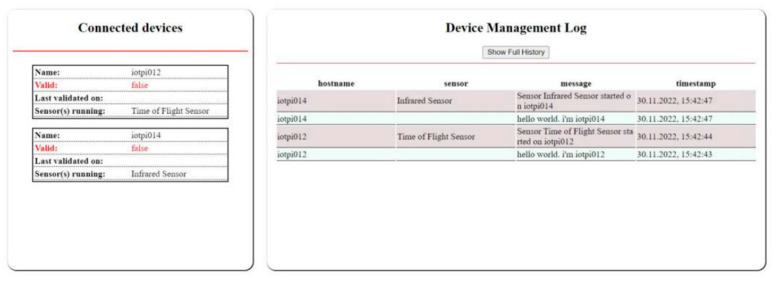
Sample of the status of each IoT device (**left**) and log messages from connected devices (**right**). The red font is used to emphasize that validity check has failed.

Two different user interfaces, as illustrated in Figure 5, were used to visualize various information related to the IoT devices and collected data. The first user interface displayed information about the IoT devices, such as their type and ID as a prefix, and the other displayed the data received from the devices. The PoC1 validation process in this PoC relied on sensors and a webcam. The webcam takes a picture when the information gathered from the sensors meets certain criteria in the Nokia LuxTurrim5G environment.

The sensor management system is open to extension with different types of IoT sensor devices. New IoT sensor devices are first registered with the MQTT server, and the subscribed UI elements may subsequently publish the data retrieved from the sensors. A sample of the device management log file is presented in Figure 5 (right). The Node.js listens to the MQTT messages and sends the data to the respective clients through WebSocket, after which the JavaScript-based Pug template engine is used to visualize the data in HTML. 

**Figure 6:**
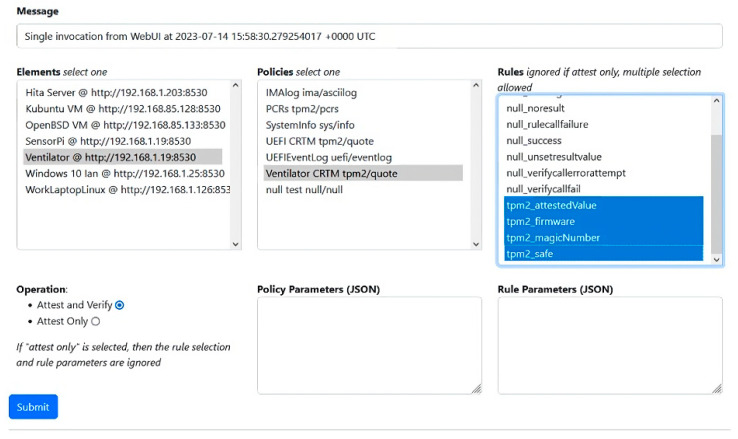
Sample of attestation management for PoC1.

The system implementation consists of devices and sensors, attestation management (Figure 6), and management UI. The software used to implement the system comprise Python 3, Vim, MQTT, Flask, and IoT device firmware. User interfaces are based on HTML, CSS, JavaScript, Pug, WebSocket, and Node.js. 

**Figure 7:**
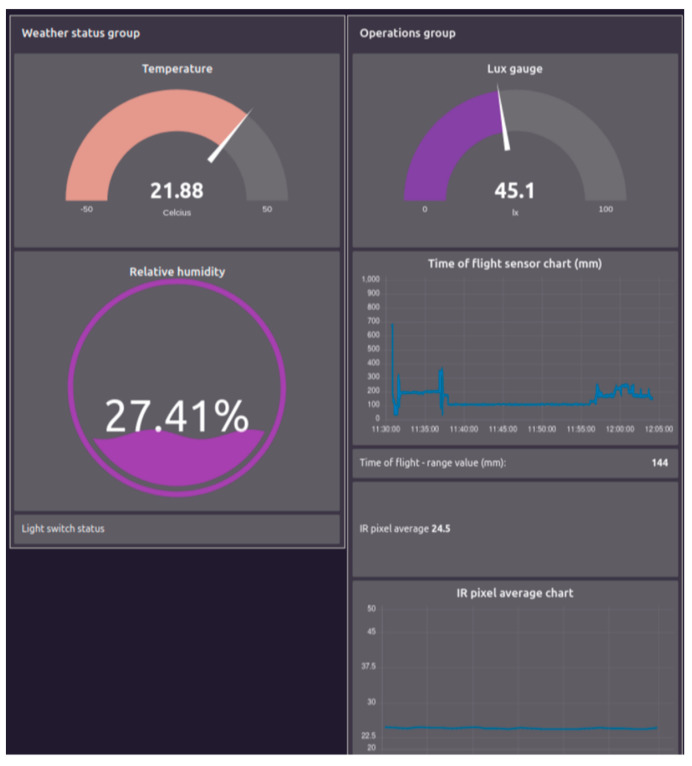
Sensor data visualization including temperature, humidity, illumination, distance (time of flight), and IR camera heat measurement.

Additionally, the data collected using the sensors are visually presented with the help of Node-RED, as depicted in Figure 7. The figure displays temperature, humidity, and illumination measurements, along with the time-of-flight sensor data that indicate the distances, and the IR pixel value displaying the average of the apparent temperatures of the infrared camera pixels.

### 5.2. PoC 2: Train Information Display Board System

This PoC project aimed to design and implement a train information display board system. The system provides the possibility of handling multiple displays at various train stations. These displays can be, for example, a central display that shows information about all trains departing from the station or displays on individual platforms. The displays can be configured with arguments to specify what information is to be shown, for example, arrivals and departures of long-distance trains and regional commuter trains.

**Figure 8:**
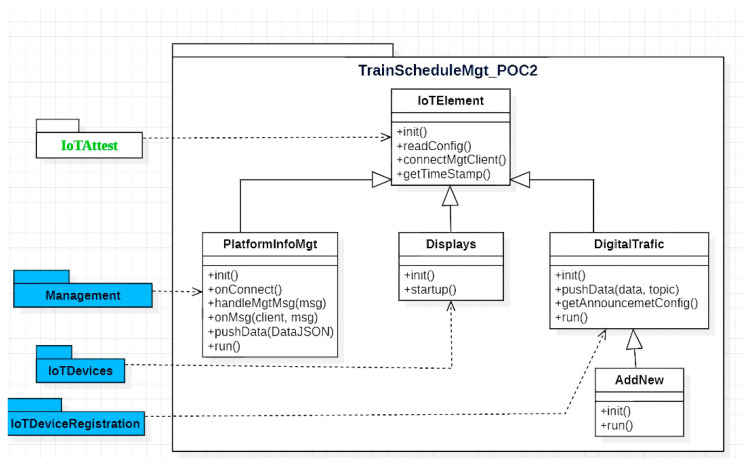
Architecture of the train information display board system.

**Figure 9:**
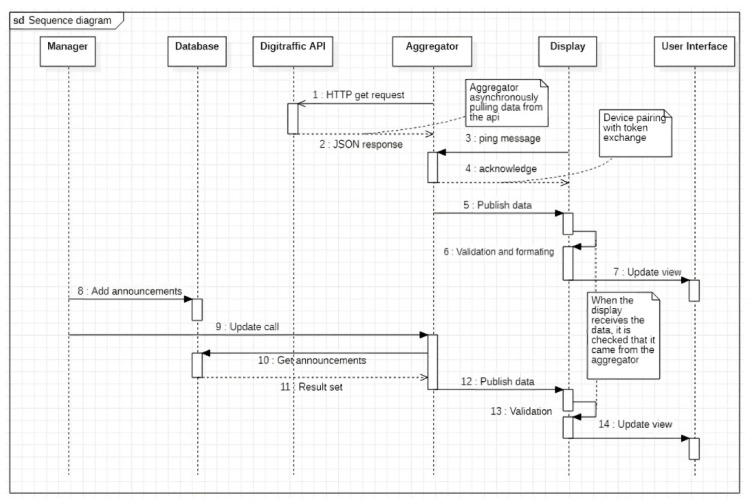
The sequence diagram of the PoC2 interaction.

Figure 8 presents the overall architecture of the train information display board system. The data aggregation component (aggregator in Figure 9), which is part of the management layer, retrieves the rail traffic data from a public API provided by Digitraffic. These data are then disseminated to various channels using the MQTT protocol, which railway station displays can subscribe to. Following subscription, the data undergoes validation and formatting before they are presented through a graphical user interface on the displays. 

The diagram presented in Figure 9 shows a sequence of events demonstrating the interaction between various entities within the system. The system consists of six main components: display, manager, aggregator, MQTT communication protocol, data validation module such as TPM or SSL/TSL, and Digitraffic open data API for train schedules. 

**Figure 10:**
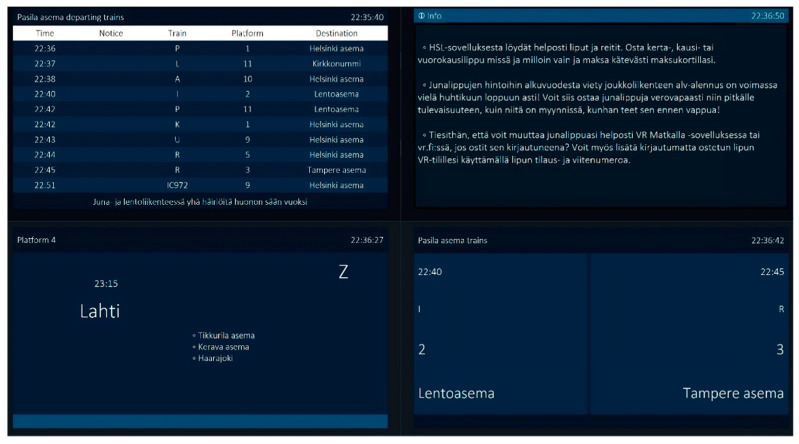
Sample of main displays and customized displays: the main monitor listing departing trains at Pasila station (top-left), a monitor providing customized information about purchasing tickets (top-right), a monitor presenting customized information about commuting to the city of Lahti (bottom-left), and a monitor showing customized information on a specific platform for trains to the airport (Lentoasema) and the city of Tampere.

The aggregator obtains data from Digitraffic through an HTTP request, which results in the retrieval of a JSON file as a response. This operation is carried out asynchronously, leading to the publication of data regardless of whether any display is active or not. Subsequently, the display is triggered to commence its operation by a ping request to the sender or client that contains a public key. This key is employed to facilitate the identification of the display by the aggregator. After receiving a message, the aggregator sends a response with its public key as a confirmation. Afterward, the aggregator shares the collected train data in their respective topics on the MQTT server. The display confirms that the data originate from the data aggregator and are not from an external source. Then, the data are formatted in a way that the user interface can show them. As the formatted data are received, the user interface updates its display accordingly. A sample of the customized content for a specific display is presented in Figure 10, which depicts four displays showing standard or customized information.

**Figure 11:**
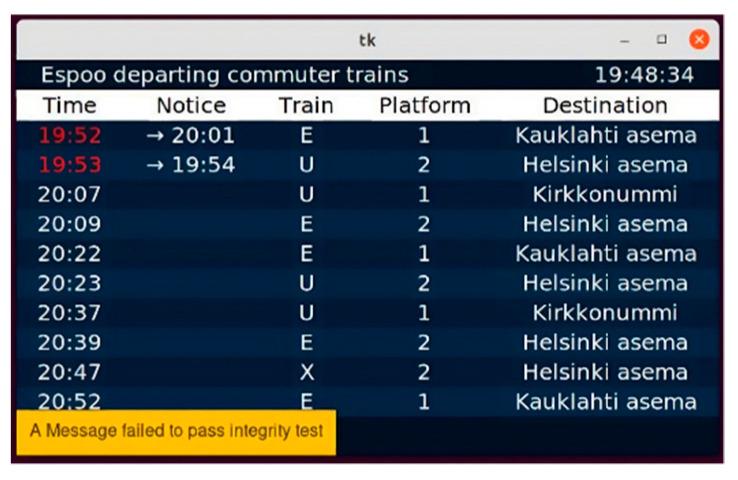
A failure notification when the integrity of the message was tampered.

Validation and the integrity of data transmitted over the system were key features of the IoT framework. Since the communication between MQTT, aggregator modules, and displays is ensured, the use of TPM is recommended to secure the messages between different entities. In this project, the developers worked remotely using a virtual development environment, and the TPM only worked as a physical device; therefore, using the TPM was inconvenient for the PoC development. Therefore, the project team applied the OpenSSL digital signature to mimic a TPM. Hence, a digital signature is added to the train traffic data sent by the aggregator program to authenticate them. The authentication takes place when the device receives a record from the MQTT channel, the encrypted content of the signature is opened, and an X509 certificate is issued, which contains the public key from the previously obtained pairing. The signature is then compared with the content of the message. If they match, we can verify the integrity of the received data. Figure 11 shows an example of a failure message. We can check the validation by capturing the record from the topic channel, falsifying its content, and sending the information directly to the MQTT topic channel subscribed to by the display. The result is that the display detects a problem in the integrity of the message and automatically discards the information. 

**Figure 12:**
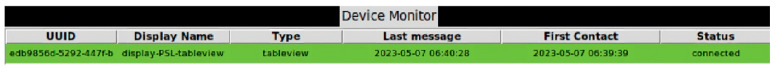
Sample of the aggregator’s log file of the display states.

**Figure 13:**
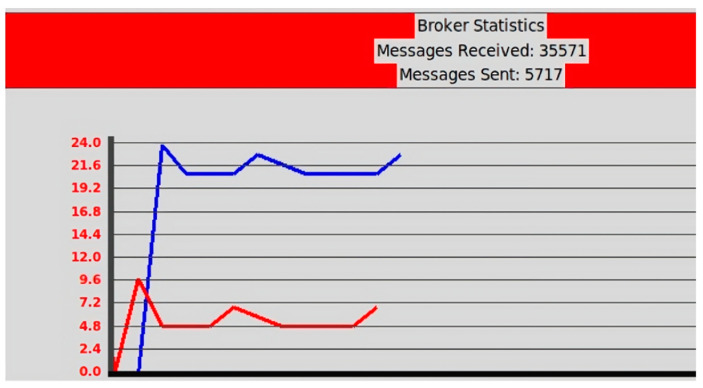
Sample of the aggregator user interface (MQTT broker statistics). The blue and red lines indicate the sent and received messages, respectively.

**Figure 14:**
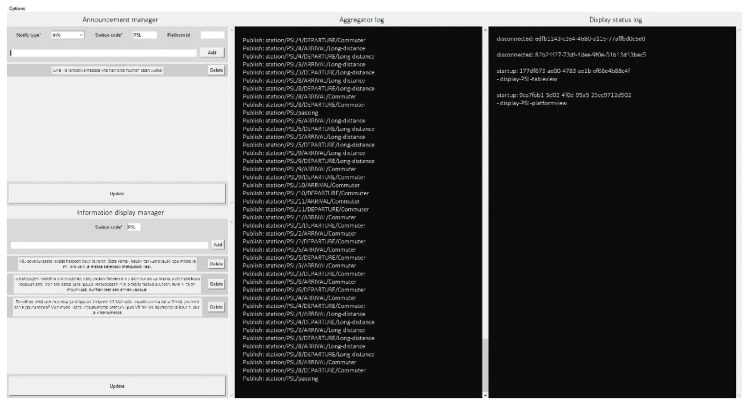
Sample of attestation management for PoC2. The message under Announcement manager informs about disruptions in train and air traffic due to bad weather. The Finnish messages under Information display manager provides advertising information about the “VR Matkalla” app and “Junalähdöt” scheduling service that provide scheduling and real-time information services for train commuters.

As Figure 12 shows, the aggregator has its own user interface consisting of log information that keeps track of the events in the system, devices, and the states of the events. The green background color indicates that the display is currently paired and connected to the aggregator, whereas the orange background color indicates that the display has sent messages that failed the integrity test. Additionally, the aggregator log file keeps statistics of sent and received messages via the MQTT broker, as shown in Figure 13. The figure indicates the number of incoming and outgoing messages in the displays. Finally, Figure 14 shows a screenshot of the attestation management of PoC2.

## 6. Questionnaire Results

The goal of the questionnaire was to gather the perceptions of the developers regarding the IoTAttest platform that they used as the basis of their development work. The questionnaire was administered to the project workers via email after their work finished. Three members of the PoC1 project group and all four members of the PoC2 project group provided answers to the questionnaire. To analyze the answers, we categorized them into three themes related to the framework and its usage from the developer’s perspective: usability and limitations; scalability, extensibility, and potential; and security and integrity. The results are presented in the following subsections according to these themes. 

### 6.1. Usability and Limitations

A majority of the developers in both groups considered the framework to be easy to use with only one respondent criticizing the lack of clear TPM instructions. The following comments from two developers emphasize IoTAttest’s ease of use and flexibility, whilst also noting a perceived difficulty with TPM:

“*I thought it was easy to use and simply implemented. Its use could suit all kinds of IoT projects well.*” (PoC1)

“*MQTT is easy but TPM is difficult to use without proper instructions.*” (PoC2)

“*The template is very easy to understand and to implement as boilerplate. Once complexities arise, the template begins to change.*” (PoC2)

Furthermore, developers believe the framework saves development time as it is based on standard, well-documented protocols and MQTT, which provides efficiency and flexibility:

“*I think it is pretty easy to use. Much of the installation process is automated with installation scripts, and the adding of new sensors to the system is a simple procedure.*” (PoC1)

“*Its ease of use saves time in development, as system planning is faster and it is easy to find documentation online.*” (PoC2)

“*It can facilitate very well, since the standardized protocols and flexible architecture of MQTT allow increased scalability and good security with help of TPM.*” (PoC1)

TPM was recommended to be used for secure authorization and attestation of IoT devices in the IoTAttest framework. However, it is not the only method available for this purpose. The developers of PoC2 faced significant challenges when trying to implement the TPM functionality, and justified the reasons as to why they resorted to utilizing SSL instead of TPM, as the following comments illustrate:

“*There were challenges in using the framework’s TPM, so we used OpenSSL identification instead.*” (PoC2)

“*Data parsing and data validation were two most complicated issues. It was difficult to integrate TPM2.0 into our virtual development environment, so we made our own solution, the SSL Digital Signature, using X509 certificates.*” (PoC2)

These comments show that although the IoTAttest framework proposes certain approaches to achieve data and device integrity, they are not set in stone and customization can be performed as per the development requirements.

The developers of PoC1 managed to fully implement their system based on the IoTAttest, also including the TPM module. The following excerpt from an answer of a PoC1 developer states that their only major issue after initial system set up was the attestation database, which was only used but not developed by the developers:

“*Once we got it running, really the only thing that caused problems was its reliance on the attestation database. Sometimes it had to be restarted. You have to be mindful that all the parts of the system are working correctly.*” (PoC1)

One potential limitation pointed out by the developers of PoC1 was related to the MQTT broker’s availability and correct operation. The following comments suggest that MQTT could become a bottleneck of the system if it fails to meet its operational expectations:

“*MQTT is dependent on the broker, so messages may not be forwarded if there is something wrong with it.*” (PoC1)

“*The entire framework relies on the MQTT module to work. If it fails or experiences downtime’, the whole system will be affected.*” (PoC1)

This limitation can be mitigated by load balancing using MQTT clustering, which is supported by some MQTT broker providers.

### 6.2. Scalability, Extensibility, and Potential

We asked the developers to provide insights on how scalable they think the IoTAttest framework is. The implemented projects were small-scale and some of the developers expressed uncertainty about the scalability of IoTAttest, as the following excerpt from a developer of PoC2 illustrates:

“*It remains to be unclear how well the framework can scale as the number of devices and data sources increase. As the system grows it may become challenging to manage.*” (PoC2)

However, the questionnaire results also indicate that several developers considered the use of MQTT to be a good choice for large IoT systems, as it is fast, efficient, and scalable. Moreover, the use of TPM and the attestation were also found to contribute to the scalability of the system. The following comments from the developers illustrates these findings:

“*The MQTT protocol works quite well if it were a very large system, thanks to its small resource constraints.*” (PoC2)

“*The MQTT allows for very easy scalability in the messaging. With naming of topics, you can add new subtopics with ease.*” (PoC1)

“*It is very scalable. The MQTT protocol is by design very flexible. And the TPM’s used along with the attestation engine is also*” (PoC1)

Some developers suggested that scalability and extensibility to new types of sensor devices are among the strengths of IoTAttest. In particular, we could find many developers expressing the view that the ability to flexibly add new devices to the system contributes to its extensibility. Moreover, one developer saw opportunities for customization and extension, and even replacement of modules of the system, thus further extending its potential usage cases. These aspects of scalability and extensibility are illustrated in the following developer quotes:

“*The scalability of the system is one of its strengths. Adding new sensors is easy with the library we made.*” (PoC2)

“*Some of the modules can be customized or even replaced, like we did by replacing TPM integrity Module with SSL Digital Signature Module (while staying true to the TPM approach as much as possible). MQTT Server can be easily extended to handle additional channels and additional modules. Management Module could be extended by splitting its functionality into separate modules. The framework could be easily extended to support additional languages and new types of devices.*” (PoC2)

The focus of the proposed IoTAttest framework was on device and data integrity. Therefore, it did not cover deeply important security aspects such as encryption, user authentication, and intrusion detection. This was also noted by PoC1 developers, one of whom recommended to add message encryption and access control to the framework to make it a more comprehensive IoT security framework, as the following quote demonstrates:

“*Currently the information needed for the attestation moves as plain text in the MQTT channel, and is thus vulnerable to attack. Encrypting the info inside a JWT-token will increase the security of the system.*” (PoC1)

Moreover, the questionnaire answers revealed that the developers suggested adding further modules related to blockchain, data analytics, and machine learning to IoTAttest to make the framework more secure and able to execute various data analytics tasks:

“*A blockchain module could be added to the framework to make it more secure.*” (PoC1)

“*The IoT Framework template does not offer any Big Data Analytics (BDA) modules, other than adding data to SQL tables. One module could be added to enhance its functionality is a Machine Learning Module.*” (PoC1)

### 6.3. Security and Integrity

The last theme of the questionnaire comprised questions regarding security and integrity aspects of the framework. The results show that developers in both groups believe that the use of TPM along with a centralized MQTT broker ensure the security of the platform, as the following comments illustrate:

“*TPM or any other module-to-module identification works fine. Also, MQTT is one of the best messaging protocols in terms of security when all data is transmitted only through one broker.*” (PoC2)

“*The MQTT provides support for authentication and encryption. Combined with TPM it can be well secured.*” (PoC1)

“*Using TPM greatly increases security. We can build apps or devices which can be attested in every circumstance. This way we know if the software/device has been tampered with. PoC1 developer.*” (PoC2)

One PoC1 developer demonstrated a correct understanding of the underlying security architecture regarding MQTT and its consequences for ensuring security and data integrity:

“*MQTT enables the use of TLS/SSL to encrypt communication between devices, which prevents eavesdropping and data modification.*” (PoC1)

Furthermore, the framework’s configurability and remote attestation allows for the detection of any changes in the device configuration, as illustrated by the following comment from a developer:

“*The framework allows the system operators to see if the devices in the system have been tampered with or if changes have been made to their original setups. This way they can see if the data that a particular sensor provides is reliable (the calibration of the sensor etc. notwithstanding).*” (PoC1)

According to the developers, major security concerns are related to MQTT messaging, physical tampering, and lack of validation of open data sources. The following quotes demonstrates these aspects:

“*MQTT messages are vulnerable to spy/other attacks.*” (PoC1)

“*Physical threat to devices would be a security issue. E.g., someone walking to a device and doing something to it. The use of open data is also hard to check to be correct.*” (PoC2)

These are potential threats to security and data integrity; thus, they are among aspects to be considered in a future version of the IoTAttest framework.

## 7. Discussion

In this paper, we proposed the concept and reference architecture of IoTAttest, an IoT security framework for facilitating the development of IoT systems that focus on data and device integrity. We then validated IoTAttest through the development of two distinct PoC IoT systems by university student groups. The objective of these projects was to utilize IoTAttest and assess its functionality from the developers’ perspective. The first PoC utilized IoTAttest to develop a sensor management system using a Raspberry Pi with sensors attached. The second PoC applied the framework to reading data from open data sources and tailoring it depending on the connected devices. The validation of the framework through PoCs demonstrated that the framework can be used as the basis for developing different types of IoT systems that have hardened data and device integrity. 

### 7.1. Answer to the Research Question

The research question was this study was: what are the key components necessary for ensuring data and device integrity in an IoT system? Through the framework development and subsequent validation, we identified the following key components for achieving data and device integrity in the IoT context: (i) an attestation component (e.g., TPM) on each IoT device that can be used to verify the device’s identity; (ii) an attestation server instance that orchestrates the IoT device attestation; and (iii) an efficient, fast and secure communication method between the components (e.g., MQTT). Additionally, the proposed framework architecture comprises other key components related to device registration, data collection, data publishing, data aggregation, and user interfaces that can be utilized as the basis of IoT system designs. 

### 7.2. How does IoTAttest Facilitate IoT Device and Data Integrity?

There are many key components involved in maintaining the integrity of IoT devices and the data that they collect. The role of these components is to ensure the security and integrity of the IoT system, the exchanged data, and the devices among different entities within the IoT system. The IoTAttest system handles the authentication and configuration of the devices and attached resources (Figure 1). The configuration is performed in accordance with the requirements of the IoT system, which includes updating the firmware of the devices. The encryption of the data transmitted between the system components via MQTT further protects the confidentiality and integrity of the devices and data. Another important aspect is access control, which makes a decision to grant or reject a device’s request to access specific data through an MQTT communication channel over a secure communication protocol such as TLS. Finally, the monitoring system on the User Interface Layer (Figure 1) enables the follow-up and monitoring of log files, IoT device performance, network traffic, and any other information that can be used to inform the user about suspicious device behavior or activities. 

### 7.3. PoCs’ Compliance with the IoTAttest Reference Architecture

Throughout the development of the PoC1 and PoC2 projects, we instructed the teams to make sure that IoTAttest is applied appropriately to ensure the integrity of the IoT devices and data in the IoT system. The overall outcomes of the PoC systems demonstrate fairly good compliance with the initial system requirements. However, there are differences in the implementation of the components. The developers reported some challenges during the implementation of the IoTAttest components. Of the two main components, MQTT and TPM, the latter posed difficulties as the developers did not have prior experience with it. Consequently, the configuration was time-consuming and the developers of PoC2 ended up using OpenSSL as an alternative solution. Unlike the TPM technology, the developers expressed the view that MQTT was easier to use, so it was extensively used in both PoC systems.

### 7.4. Scalability and Extensibility of IoTAttest

The scalability of the IoTAttest framework driven by the MQTT protocol is considered as one of its strengths. Although we did not conduct scalability evaluation in this study, other research has evaluated various MQTT broker solutions, suggesting that some of them are highly scalable [47]. As the developer questionnaire results in Section 6.2 demonstrate, the PoC developers also found the potential scalability of the IoTAttest framework to be high due to the use of MQTT. Similarly, extensibility is another strength of IoTAttest, as the architecture was designed to allow easy registration of new IoT devices. This was also identified by the developers through their questionnaire answers that suggested various ways in which the framework could be extended and adapted, such as by adding new types of sensor devices (both real and virtual), replacing TPM by another technology like SSL digital signatures, and adding new languages. Overall, the results of the validation via PoC development suggest that IoTAttest has significant potential to be used in various IoT applications, although the scalability remains to be evaluated in a future study.

### 7.5. Comparison of IoTAttest with Other Frameworks

We compared the features of IoTAttest and other attestation frameworks for device and data integrity. The results of the comparison are summarized in Table 3 and the other IoT attestation frameworks are briefly described below. The content of the table is based on the review of the IoT attestation frameworks’ descriptions. Therefore, we have not tested the other frameworks using simulations. 

Abera et al. [48] proposed the DIAT system to verify the correctness and the control-flow of the data for attestation. DIAT ensures that the data sent are not maliciously changed during transportation, generation, and processing. Abera et al. [48] validated their DIAT system through a simulation environment with a remote run-time attestation scheme that allows for the provision of authentic integrity data exchange. The use of static (binary) attestation is not often recommended since it prevents the detection of run-time attacks. Therefore, a run-time attestation schema is more recommended. Aligned with this, Moreau et al. [49] proposed a continuous remote attestation framework for IoT (CRAFT) which is aimed to be a general solution to IoT attestation that supports different attestation protocols. The conducted performance evaluation showed little or no overhead. Lastly, US-AID is an attestation system that provides collective attestation schemas for autonomous dynamic network environments comprising embedded devices, where the attestation is implemented through key exchange [50]. The system was validated through a PoC implementation and subsequent simulation to assess its scalability and practicality.

### 7.6. Recommendations for IoT System Development Work

The framework validation via PoC development projects indicates that the framework can be efficiently used to develop IoT systems with device and data integrity. Moreover, by implementing two different IoT systems based on the framework, we showed, to some extent, its scalability and flexibility based on the nature of the IoT system requirements. From a software engineering perspective, our experience suggests that successful project delivery within designated budgets heavily relies on a solid design. This includes envisioning potential solutions and clearly defining and listing use cases. Before implementation, high-level UML architectural designs are approved by stakeholders, such as the product owner and scrum master. Discussions on the development environment and the technologies to be used are finalized with the product owner prior to implementation.

Our experiments have shown that an agile approach was highly efficient in utilizing the proposed IoTAttest framework. Therefore, applying proper project management methods ensures successful delivery even for complex IoT systems.

### 7.7. Implications of the Results

As the number of IoT devices and manufacturers in the market is growing, it is likely that the number of security breaches will also increase. Using firewalls and intrusion detection systems are amongst popular methods in safeguarding an IoT system against external threats. Similarly, providing device and data attestation services in an IoT system adds another level of security so that people who need the data, particularly in critical systems, can trust that they come from an authorized source and have not been tampered with. The proposed IoTAttest framework can be utilized as a starting point for developing secure IoT systems from the perspectives of data and device integrity. Therefore, it can speed up the development of IoT systems and increase their overall security level.

The further development of the framework has been influenced by input from the PoC development teams; however, the details about this development are beyond the scope of the paper. It should be noted that the PoC applications have also contributed to the advancement of training materials for universities, including those in Finland, as well as internal Nokia training programs. Moreover, we have included online references to TPM commands provided by Nokia and resources available through the tpm.dev and tpm2tools communities.

### 7.8. Limitations

The primary objective of this study was to formulate and construct an IoT security framework while showcasing its practical application through real-world PoC projects. Consequently, we pinpoint certain limitations of the study as follows. 

First, the IoTAttest framework has insufficient support for developers in terms of documentation. One of the primary challenges faced by the PoC teams was the configuration of the TPM. The PoC1 team was able to successfully configure and apply the TPM to their project. However, despite extensive efforts, the PoC2 team encountered difficulties in utilizing the TPM. As a result, they had to employ an alternative solution (OpenSSL) to avoid project failure and meet their schedule. The main limitation that led to this decision was the lack of sufficient experience and resources specifically related to TPM configuration.

The second limitation of the study’s results is the absence of security evaluation. We did not include any reports on security tests conducted on the IoT framework or the developed PoCs, such as system hacking or system attacks. The main focus of this paper has been on outlining the functionalities of the framework and demonstrating its feasibility through the PoCs. However, it is worth noting that there are plans to address the security perspective of the framework in a separate study.

Third, the first version of the IoTAttest framework focuses solely on device and data integrity through attestation. Therefore, it does not cover important aspects such as firewall configuration, blockchain for data integration, intrusion detection and prevention, user authentication, details of strong encryption, and so forth. We acknowledge the importance of these aspects and plan to elaborate on them in future work.

Fourth, only two IoT system projects were created based on the IoTAttest framework under the supervision of the second author who proposed the framework concept. It would also be essential to evaluate the usability of the framework with independent developers who only utilize the documentation provided with the framework.

Fifth, we analyzed the framework’s effectiveness based on a survey answered by the developers. However, the effectiveness of IoTAttest also needs to be verified through simulations in future work. 

## 8. Conclusions

The advancement of technology has made the design and development process of IoT systems and services easier. However, this advancement often brings along a set of challenges that designers and developers must address, particularly concerning the security, privacy, and overall trustworthiness of the system. As a result, a security framework must often undergo updates to effectively mitigate security constraints and evolving requirements. This also holds true in the context of IoT security frameworks. Here the framework must adhere to industry standards but also continuously evolve to cover a broader range of application contexts while maintaining robust security measures. In this study, we introduced IoTAttest, a security framework for IoT application development that focuses on data and device integrity, which leads to increased privacy preservation and trustworthiness. The proposed framework includes the essential components that need to be considered for the implementation data and device integrity in IoT systems: an attestation component (e.g., TPM) on each IoT device that can be used to verify the device’s identity; an attestation server instance that orchestrates the IoT device attestation; and an efficient, fast and secure communication method between the components (MQTT). IoTAttest enables system design for security, privacy, access control, monitoring, and incident response planning and reporting. Other contributions of this study in addition to the open and extendable IoTAttest framework include a validation of the framework through the implementation of two real-world PoC systems based on IoTAttest, a subsequent qualitative evaluation by surveying the developers’ experiences and perceptions concerning the use of IoTAttest, and a comparative analysis of IoTAttest and other IoT attestation frameworks. 

Despite the limitations expressed in Section 7.6, the IoTAttest framework holds promise in enabling secure and efficient IoT system implementations. It provides a solid foundation for organizations to build upon, ensuring that security is embedded at every level of the IoT ecosystem. As IoT technologies continue to evolve, it will be crucial to address the identified limitations and further refine the framework to meet the current and emerging IoT security challenges. 

In our forthcoming research endeavors, we intend to comprehensively evaluate IoTAttest from the standpoints of physical and cyber security. Our future work objectives encompass thorough simulation tests for a comparative analysis of the IoTAttest alongside established frameworks within the domain. Additionally, we envision the incorporation of the blockchain technology within the IoTAttest framework, with a focus on gauging enhancement in the security and privacy of exchanged data. 

## Figures and Tables

**Table 1:** The applied IoT devices and software in the PoC1 project.

No:	Device/Software	Comments
1	Raspberry pi	Raspberry Pi 4 model B
2	ToF-Sensor	Time-of-flight sensor
3	Temperature and Humidity sensors	DHT11
4	Python	Python3
5	MQTT	MQTT server for channel management
6	Flask	For implementation of attestation, web-camera access, and REST
7	TPM 2.0	Security key for attestation using Nokia Attestation Engine
8	Attestation server	Nokia Attestation Engine A10

**Table 2:** The applied IoT devices and software in the PoC2 project.

No:	Device/Software	Comments
1	Raspberry pi	Raspberry Pi 4 model B
2	Monitors	4 × 24′ displays.
3	PIS	Platform Information System.
4	Python	Python3
5	MQTT	MQTT server for channel management
6	Flask	For implementation of attestation, web-camera access, and REST
7	TPM 2.0/OpenSSL	Security key for attestation using Nokia Attestation Engine
8	Attestation server	Nokia Attestation Server A10

**Table 3:** Comparison of features of IoT attestation frameworks.

Features	IoTAttest	DIAT [48]	CRAFT [49]	US-AID [50]
Year	2023	2019	2021	2018
Static Network	Y	N	N	Y
Attestation Spread	Device-to-Device	Device-to-Device	Device-to-device	Device-to-Device
Unlimited network lifespan	Y	Y	Y	N
Heartbeats	Y	Y	Y	Y
Openness	Y	Y	Y	Y
Attestation Engine	Nokia Attestation Engine A10	No	Remote Attestation protocol (open)	Collection Attestation (In-network Attestation)
Message relay technology	MQTT	PEP/MQTT	No specific message relying	-
Identification technology	TPM 2.0	No specific identification	TPM	Combination of In-network and key exchange mechanism and proofs-of-non- absence.
Validation method	Two PoC implementations	PoC (smart home)	Simulation	PoC (6 drones)

## Data Availability

Data presented in this study are available on reasonable request from the first author.

## Appendix A. Questionnaire for the Developers

The developers were asked to answer the following questions, in addition to demographics information, after finishing their projects.

*Usability and limitations:*
  - What do you think about the ease-of-use of the IoT framework template?
  - Did you face any challenges when using the IoT framework template?
  - To what extent does the IoT Framework facilitate the work of an IoT system developer?
  - What do you think are the limitations of the IoT Framework?
*Scalability, extensibility, and potential:*
  - What do you think about the scalability of the IoT Framework template?
  - To what extent is the IoT Framework extendable?
  - Can you think of any other IoT applications where the IoT Framework could be utilized?
  - Can you think of any module that could be added to the IoT Framework?
  - To what extent can the IoT Framework be integrated with third-party tools and platforms?
*Security and integrity:*
  - Can you identify any potential reliability or security issues with the IoT Framework?
  - To what extent does the framework help increase IoT security and data integrity?
